# Supplementary material for: Neighborhood context, genetic influences, and life satisfaction: Evidence from the German twin family panel
Source: PLoS One. 2025 Aug 25;20(8):e0316416. doi: 10.1371/journal.pone.0316416 (PMC12377583; doi:10.1371/journal.pone.0316416)
Supplement: Attachment — Submitted filename: Report.pdf [file pone.0316416.s001.pdf]

PONE-D-24-57036

Neighbourhood Deprivation, Genetic Predisposition, and Life Satisfaction: Evidence from the German Twin Family Panel

---

Interesting and excellent study.
Overall, the study is important and contributes to the genetic and psychology research area. There are some suggestions and comments as below.

(1) The study is focusing on gene-environment interactions (GxE) on individual life satisfaction. Participants between 10 and 24 years old. There is a big gap in individual developmental stages.
From a psychological view, it involved the development from early adolescence to young adults. Many other factors (including developmental tasks) will influence individual life satisfaction. Kindly justify.

(2) Life Satisfaction—Instrument
a) How many total items were used for data collection?
b) What do you mean by "*comprising five items each*"?
c) How does CFA use to measure satisfaction level? Any data for CFA? The below statement is a bit confusing. Kindly revise and justify.

"The final life satisfaction score was created using confirmatory factor analysis of the raw indicators" .

(3) Methodology – Data Collection.
a) Kindly justify when the data collection is done. The below statements are not reported deeply.

"The data were accessed for research purposes on May 2024."
"Data collection for the Face-to-Face (F2F 1) survey occurred in two phases: Subsample A was collected between September 28, 2014, and May 28, 2015, while Subsample B was collected between September 16, 2015, and April 18, 2016"

(4) Family SES is measured as a factor based on parental years of education, occupational status (ISEI), and OECD household net income. However, it is not reported clearly in the main document. Kindly justify and report the data for the below statements:

" .. in the statistical analysis we additionally control for family SES, measured as a factor based on parental years of education, occupational status (ISEI) and OECD household net income. Additionally, we control for birth cohort status, using a categorical variable indicating whether a twin belongs to cohort 2, 3, or 4."

(5) Discussion lacks citation or previous study to support. Suggest discussing the findings through theories as mentioned in the subheading "Theoretical Framework.". Also, discuss the main finding and overall contribution.

(6) Suggest to add the subheading “Implication and Recommendation” – Explain the implication from a theoretical and practical view. How does this study contribute to both the genetic and psychology areas?
