# Supplementary material for: Neighborhood context, genetic influences, and life satisfaction: Evidence from the German twin family panel
Source: PLoS One. 2025 Aug 25;20(8):e0316416. doi: 10.1371/journal.pone.0316416 (PMC12377583; doi:10.1371/journal.pone.0316416)
Supplement: Attachment — Submitted filename: Response to Reviewers.docx [file pone.0316416.s003.docx]

PONE-D-24-57036

**Response to reviewers' comments on the manuscript “Neighbourhood Deprivation, Genetic Predisposition, and Life Satisfaction: Evidence from the German Twin Family Panel”**

Thank you for the opportunity to submit a revised version of our manuscript titled *“Neighbourhood Deprivation, Genetic Predisposition, and Life Satisfaction: Evidence from the German Twin Family Panel”* to *PLOS ONE*. We sincerely appreciate the time and effort that you and the reviewers have dedicated to providing thoughtful and constructive feedback. We are especially grateful to the reviewers for their insightful comments, which have significantly improved the clarity and overall quality of the manuscript. Below, we provide a detailed, point-by-point response to each of the reviewers’ suggestions

**Comment 1:**

The study is focusing on gene-environment (GxE) on individual life satisfaction. Participants between 10 and 24 years old. There is a big gap in individual developmental stages.

From a psychological view, it involved the development from early adolescence to young adults. Many other factors (including developmental tasks) will influence individual life satisfaction. Kindly justify.

***Response:***

Thank you very much for highlighting the importance of age-related differences in our analysis. We fully acknowledge that our sample spans a broad developmental range—from early adolescence to young adulthood—which may introduce variability in the GxE estimates due to age or cohort effects.

While we recognize the value of investigating these developmental factors, modeling a three-way interaction (PGS × Neighborhood × Age) would significantly increase the complexity of our analysis and likely exceed our available statistical power to yield robust estimates. For this reason, we have focused our current analysis on the overall gene-environment interaction (GxE), averaged across the age range.

We agree that exploring potential age-specific patterns in GxE interactions is an important avenue for future research and appreciate you drawing attention to this point.

**Comment 2**

Methodology – Data Collection.

Kindly justify when the data collection is done. The below statements are not reported deeply.

***Response*:**

We appreciate the Reviewer’s comment and have added a clarifying sentence under the *Participants* subheading to specify the timing and scope of data collection. See below:

“In this study, we included participants from Cohorts 2 (twins born in 2003/2004), 3 (twins born in 1997/1998), and 4 (twins born between 1990 and 1993) who participated in both the first (F2F1) and third (F2F3) face-to-face waves of the TwinLife study and who provided saliva samples during F2F3. The F2F1 wave was conducted between September 2014 and May 2015 (Subsample A), and between September 2015 and April 2016 (Subsample B). The F2F3 wave took place from November 2018 to July 2019 (Subsample A), and from September 2019 to June 2020 (Subsample B).”

**Comment 3:**

Family SES is measured as a factor based on parental years of education, occupational status (ISEI), and OECD household net income. However, it is not reported clearly in the main document. Kindly justify and report the data for the below statements:

***Response*:**

We thank the Reviewer for this comment. In response, we have added a description of the confirmatory factor analysis model fit and reliability indices for Family SES in the *Covariates* subheading.

“The CFA model for family SES was saturated, yielding perfect fit indices: 𝜒^2^(0) = 0, 𝑝 = NA, RMSEA = 0, CFI = 1.00, TLI = 1.00, SRMR = 0. The reliability indices were: Cronbach’s 𝛼 = .40 and McDonald’s $= .70. Although Cronbach’s 𝛼 was relatively low—likely due to violations of the tau-equivalence assumption, McDonald’s 𝜔 indicated acceptable construct reliability. Given that 𝜔 does not assume equal factor loadings and is more appropriate in structural equation modeling contexts, we consider the construct to demonstrate adequate internal consistency.”

**Comment 4:**

Discussion lacks citation or previous study to support. Suggest discussing the findings through theories as mentioned in the subheading “Theoretical Framework.”. Also, discuss the main finding and overall contribution.

***Response*:**

We thank the Reviewer for this insightful comment. In response, we have substantially revised the Discussion section to more clearly engage with the theoretical framework outlined earlier in the manuscript. Specifically, we now interpret our findings through the lens of established G×E mechanisms (e.g., compensation, suppression) and neighborhood effects theories (e.g., collective efficacy, institutional resources), supported by relevant literature (e.g., Shanahan & Hofer, 2005; Sampson et al., 1997; Ellen & Turner, 1997). We also explicitly highlight the main findings and articulate the overall contribution of the study—namely, extending G×E research to the domain of life satisfaction and contextualizing genetic influences within varying neighborhood conditions. These revisions are reflected in the revised Discussion section of the manuscript.

**Comment 5:**

Suggest to add the subheading “Implication and Recommendation” – Explain the implication from a theoretical and practical view. How does this study contribute to both the genetic and psychology areas?

***Response*:**

We appreciate the Reviewer’s suggestion to include a dedicated subheading on Implication and Recommendation. In response, we have added a new section that outlines both the theoretical and practical implications of our findings. This section highlights the study’s interdisciplinary contribution—bridging behavioral genetics and psychological research on subjective well-being—while also emphasizing how neighborhood-level interventions may moderate genetic risks or support genetic potentials. The new section is titled Implication and Recommendation and can be found following the Discussion.
